# Epidermal growth factor potentiates *in vitro *metastatic behaviour of human prostate cancer PC-3M cells: involvement of voltage-gated sodium channel

**DOI:** 10.1186/1476-4598-6-76

**Published:** 2007-11-24

**Authors:** Pinar Uysal-Onganer, Mustafa BA Djamgoz

**Affiliations:** 1 Neuroscience Solutions to Cancer Research Group, Division of Cell and Molecular Biology, Sir Alexander Fleming Building, Imperial College London, South Kensington Campus, London SW7 2AZ, UK; 2 Prostate Cancer Research Group, Division of Surgery, Oncology, Reproductive Biology and Anaesthetics, Faculty of Medicine, Imperial College London, Hammersmith Hospital, Du Cane Road, London W12 0NN, UK

## Abstract

**Background:**

Although a high level of functional voltage-gated sodium channel (VGSC) expression has been found in strongly metastatic human and rat prostate cancer (PCa) cells, the mechanism(s) responsible for the upregulation is unknown. The concentration of epidermal growth factor (EGF), a modulator of ion channels, in the body is highest in prostatic fluid. Thus, EGF could be involved in the VGSC upregulation in PCa. The effects of EGF on VGSC expression in the highly metastatic human PCa PC-3M cell line, which was shown previously to express both functional VGSCs and EGF receptors, were investigated. A quantitative approach, from gene level to cell behaviour, was used. mRNA levels were determined by real-time PCR. Protein expression was studied by Western blots and immunocytochemistry and digital image analysis. Functional assays involved measurements of transverse migration, endocytic membrane activity and Matrigel invasion.

**Results:**

Exogenous EGF enhanced the cells' *in vitro *metastatic behaviours (migration, endocytosis and invasion). Endogenous EGF had a similar involvement. EGF increased VGSC Nav1.7 (predominant isoform in PCa) mRNA and protein expressions. Co-application of the highly specific VGSC blocker tetrodotoxin (TTX) suppressed the effect of EGF on all three metastatic cell behaviours studied.

**Conclusion:**

1) EGF has a major involvement in the upregulation of functional VGSC expression in human PCa PC-3M cells. (2) VGSC activity has a significant intermediary role in potentiating effect of EGF in human PCa.

## Background

Although prostate cancer (PCa) is the most commonly occurring cancer in males over the age of 65 [1], many problems remain in its clinical management, as regards both definitive diagnosis and long-lasting therapy [2]. A novel 'neuroscience' approach to understanding the pathophysiology of PCa suggested that upregulation of voltage-gated Na^+ ^channels (VGSCs) could be an accelerating factor in metastatic disease [3]. Thus, we have shown previously that functional VGSC expression could distinguish strongly and weakly metastatic human and rat PCa cells [4,5]. Importantly, application of tetrodotoxin (TTX), a highly specific blocker of VGSCs, suggested that VGSC activity could directly enhance metastatic ability by potentiating a range of *in vitro *cellular behaviours integral to the metastatic cascade: morphological enhancement [6], directional motility [7], secretory membrane activity [8], adhesion [9], gene expression, including auto-regulation [10] and invasion [4,5,11]. In fact, over-expression of VGSC alone was found to be "necessary and sufficient" to confer invasive potential on non-metastatic human PCa cells [12].

The catalytic/pore-forming VGSC α- subunit (VGSCα) responsible for the functional activity was found to be Nav1.7, upregulated at mRNA level by > 1000-fold in strongly vs weakly metastatic rat and human PCa cells [13]. Furthermore, VGSCα protein and Nav1.7 mRNA expression were also markedly up-regulated in human PCa *in vivo *[14]. In fact, analysis of "receiver- operator characteristics" suggested that Nav1.7 could serve as an effective functional diagnostic marker for PCa [14].

**Figure 1:**
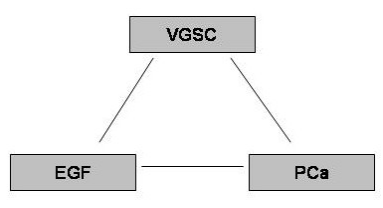
The possible triangular relationship between EGF, VGSC and PCa.

However, the mechanism(s) responsible for the functional VGSC expression in metastatic PCa is not known. VGSCs have been found to be regulated by growth factors, such as fibroblast growth factor (FGF), nerve growth factor (NGF), epidermal growth factor (EGF), in various human and rat cell lines, such as pheochromocytoma PC12 cells [15-17] and rat PCa Mat-LyLu cells [18,19]. On another front, it has also been emphasised that growth factors could play **a **major role in progression of human PCa [e.g. [20,21]]. Moreover, increased EGF expression also has been confirmed in human PCa *in vivo *[22]. Thus, there is the following possible triangular relationship (Fig. 1) and EGF could be responsible for the VGSC upregulation in PCa. This possibility has been tested in the present study using the strongly metastatic human prostate epithelial PC-3M cell model which expresses both functional VGSCs [5] and EGF receptors [23].

## Results

The overall approach was as follows: 1) Effects of exogenous EGF on PC-3M metastatic cell behaviours (MCBs) were tested; (2) possible involvement of VGSC activity in the EGF-induced effects was determined; and (3) the level (mRNA or protein) at which such VGSC involvement could occur was elucidated. The results obtained are described below hierarchically, from functional to molecular aspects.

### Effects of EGF on *in vitro *metastatic cell behaviours

**Figure 2:**
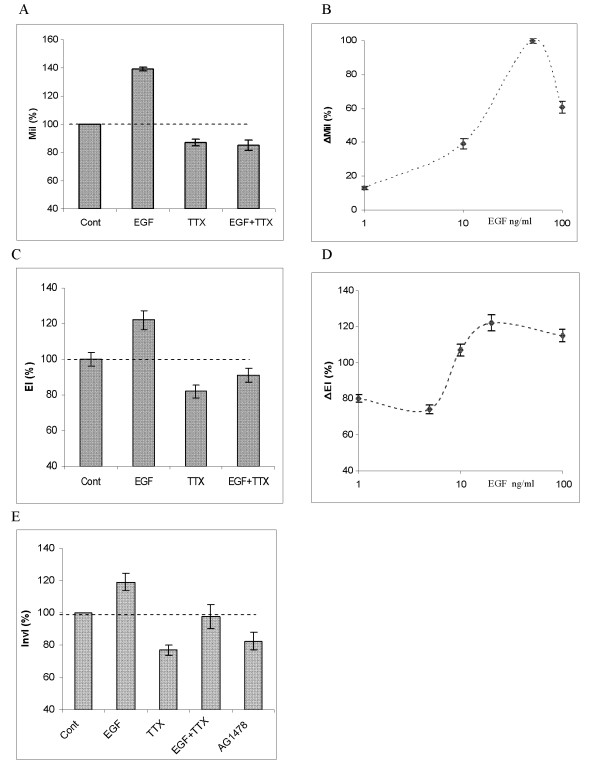
Functional evidence for EGF-induced enhancement of metastatic cell behaviours via VGSC expression/activity in PC-3M cells. (A) Migration index (MiI), expressed relative to the control level (Cont), fixed as 100 %. Effects of EGF (50 ng/ml), TTX (500 nM) and EGF+TTX are shown. (B) Dose dependence of the effect of EGF on MiI. ΔMiI denotes the percentage change (increase) in MiI induced by increasing concentrations of EGF, expressed relative to the maximum (fixed as 100 %) seen for 50 ng/ml. (C) Endocytosis index (EI), expressed as percentage of the control level (Cont). Effects of EGF (20 ng/ml), TTX (500 nM) and EGF+TTX are shown. (D) Dose dependence of the effect of EGF on EI. ΔEI denotes the change (increase) in EI induced by given concentrations of EGF. (E) Boyden chamber invasion assay data. Effects of EGF (100 ng/ml), TTX (500 nM), EGF+TTX and AG1478 (100 nM) are shown. Invasion index (InvI) denotes the percentage of cells crossing the membrane in Transwell assays. Each data point or histobar denotes mean ± standard error (n = 4).

Exogenous EGF (1–100 ng/ml) significantly increased transverse migration of PC-3M cells in a dose dependent manner (p < 0.05 for all concentrations; n = 9; Fig. 2A and 2B). The greatest effect was seen for 50 ng/ml EGF, which increased migration by 39 ± 1.2 % (Fig. 2A &2B). In most of the experiments that followed, working concentrations of EGF around this peak (i.e., 20, 50 or 100 ng/ml) were used. In endocytosis assays, treatment with EGF (20 ng/ml) enhanced HRP uptake by 23 ± 5.4 % (p = 0.01; n = 9; Fig. 2C). This effect was also concentration dependent (Fig. 2D). Interestingly, in both assays, increasing the EGF concentration ultimately produced reduced effects (Fig. 2B &2D). In Boyden chamber invasion assays, EGF (100 ng/ml) increased the cells' invasiveness by 20 ± 5.2 % (p < 0.03 cf. control; n = 4; Fig. 2E). Application of 100 nM AG1478, an inhibitor of EGF receptor, alone had the opposite effect, reducing cell invasion by 19 ± 5.4 % (p = 0.02, n = 4; Fig. 2E). This result suggested that the potentiating effect of EGF on MCBs also occurred endogenously.

All three assays were consistent, therefore, in showing that EGF enhanced PC-3M cells' *in vitro *MCBs.

### VGSC involvement in the effects of exogenous EGF

In all 3 functional assays used, application of TTX (500 nM) alone reduced MCBs: Migration (by 11 ± 2.3 %; p < 0.01; n = 6), endocytosis (by 18 ± 3.5 %; p = 0.02; n = 9) and invasion (by 19 ± 5.2 %; p = 0.04; n = 4).) (Fig. 2A, C &2E). The effects of TTX were dose-dependent (not shown). Importantly, TTX when co-applied substantially blocked the enhancement effects of exogenous EGF (20 – 100 ng/ml) in all 3 assays (Fig. 2A, C &2E). In the case of migration and endocytosis, there was a strong blocking effect of TTX on the EGF-induced enhancement; thus, for TTX vs EGF + TTX, P = 0.23 (migration) and P = 0.12 (endocytosis), ie EGF had no effect in the presence of TTX. Essentially the same effect was observed for invasion (Fig. 2E), although the difference in the values of InvI for TTX and EGF + TTX was only just not significant (P = 0.051). Thus, again, TTX blocked the effect of EGF in enhancing invasion.

It was concluded that for all 3 *in vitro *functional assays, EGF potentiated MCBs primarily via VGSC activity.

### Effects of EGF on VGSC mRNA and protein levels

**Figure 3:**
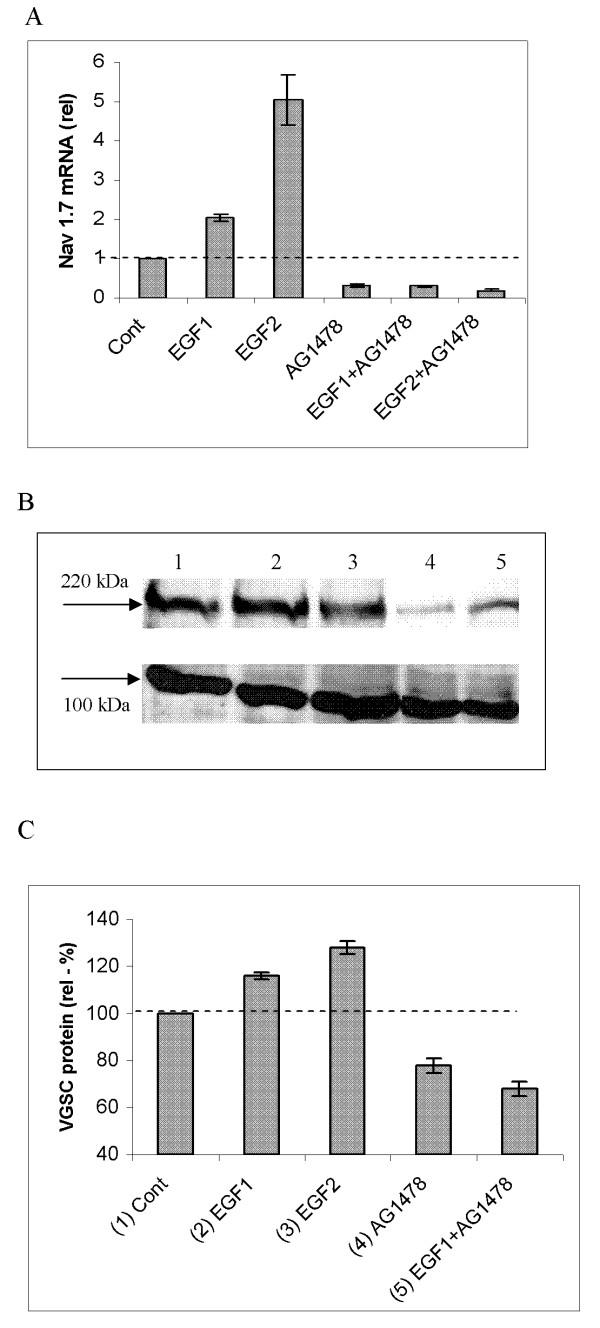
Effects of EGF on VGSC mRNA and protein expression in PC-3M cells. (A) Real-time PCR measurements of Nav1.7 mRNA expression. Data are plotted as fold-differences relative to the control (Cont) level (dotted line). All treatments were for 24 hours. Treatments were as follows: EGF1 (50 ng/ml), EGF2 (100 ng/ml), AG1478 (100 nM), and EGF1/2+AG1478. EGF significantly enhanced mRNA expression, and this was suppressed by co-treatment with AG1478 (100 nM). (B) A typical Western blot of effects on total VGSC protein expression (50 μg of protein per lane) over 24 h, with pan-VGSCα and anti-actinin antibodies. Arrows indicate molecular weights of ~220 kDa (for VGSCα) and ~100 kDa (for actinin). The lanes denote the following: 1) Control (Cont.; cells in 0.5 % FBS); (2) EGF1 (50 ng/ml); (3) EGF2 (100 ng/ml); (4) AG1478 (100 nM); and (5) EGF1 + AG1478. (C) Quantification and averaging of the data shown in (B) with the corresponding conditions (1–5). VGSC protein expression wasquantified from band optical density. Each histobar (in A and C)denotes mean ± standard error (n = 4–6).

Application of EGF (50 and 100 ng/ml) to PC-3M cells for 24 h caused significant (2- and 5.5- fold, respectively) increases in mRNA expression of Nav1.7, the predominant VGSC subtype expressed in these cells (p < 0.01; n = 4 for both concentrations; Fig. 3A). Treatment with AG1478 (100 nM) reduced the basal Nav1.7 mRNA level by 70 ± 2.1 % (p < 0.01; n = 4; Fig. 3A). Co-treatment with AG1478 abolished the effect of exogenous EGF on Nav1.7 mRNA levels (Fig. 3A).

Similar treatment with EGF (50 and 100 ng/ml) caused increases in total VGSC protein expression by 16 ± 1.5 and 28 ± 2.1 %, respectively (for both: p < 0.01, n = 6; Fig. 3B &3C). In contrast, AG1478 suppressed total VGSC protein expression by 22 ± 3.1 % (p < 0.01; n = 6; Fig. 3B &3C). AG1478 also suppressed the effect of exogenous EGF in upregulating total VGSC protein expression; there was no difference in the levels of VGSC protein expression for EGF+AG1478 and AG1478 (p > 0.05; n = 6; Fig. 3B &3C).

**Figure 4:**
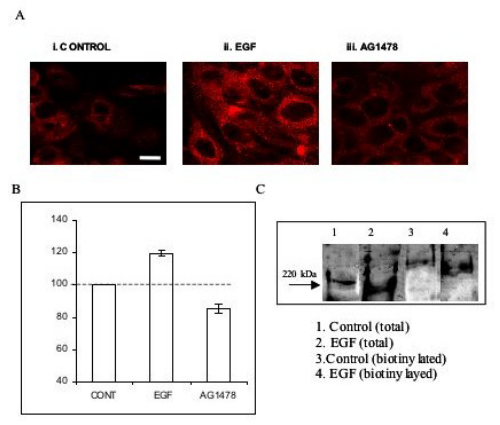
Confocal microscopy and densitometric analysis of VGSC protein expression in PC-3M cells. (A). Typical confocal images. (i) Control. (ii) EGF (100 ng/ml). (iii) AG1478 (100 nM). Each treatment was for 24 h. Scale bar, 20 μm (applicable to all panels). (B) Signal density, ie optical density of plasma membrane (PM) VGSC immunocytochemistry, corresponding to images such as (A). Each histobar denotes mean ± standard error (n = 50 cells/3 separate experiments). (C) Effects of EGF (similar treatment as in A) on total and PM VGSC expression. The PM fraction was immunoprecipitated by biotin labelling. Key: 1) Control total VGSC protein. (2) EGF-treated total VGSC protein. (3) Control PM VGSC protein. (4) EGF-treated PM VGSC protein. Note the change in the molecular size of the biotinylated fractions (lanes 3 & 4).

The effect of EGF (exogenous and endogenous) in increasing VGSC protein expression was also apparent in plasma membrane (PM). Thus, exogenous EGF (100 ng/ml) and AG1478 (100 nM) altered PM expression by +20 ± 2.0 and -15 ± 2.6 %, respectively (for both: p < 0.01; n = 5; Figs. 4Ai–iii &3B). In a preliminary experiment (n = 2), an alternative method, involving cell surface biotinylation similarly showed that EGF increased plasma membrane VGSC protein expression (Fig. 4C, lane 4 vs 3).

**Figure 5:**
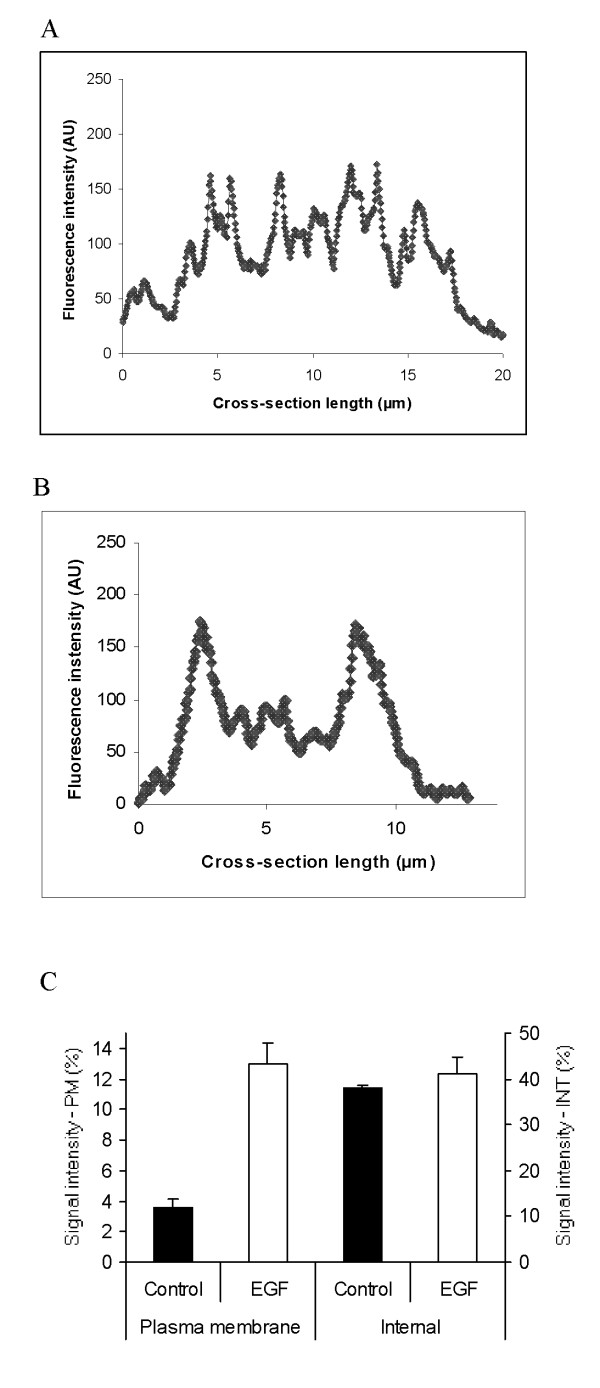
Effect of EGF on sub-cellular distribution of VGSC protein expression, determined by immunocytochemistry, confocal microscopy and digital imaging. VGSC expression was denoted by the fluorescence intensity (arbitrary units; AU) of the immunocytochemical label. (A) Typical profile of VGSC protein expression scanned across the cell. (B) Same as (A) after treatment with EGF (100 ng/ml) for 24 h. (C) Data quantified from profiles shown in (A) and (B) dividing the cellular cross-sections into cytoplasmic/internal ("INT") and plasma membrane ("PM") fractions, as described in the Methods. Each histobar denotes mean ± standard error (n = 18 cells from 3 separate experiments for each condition).

Finally, the possible effect of EGF on sub-cellular distribution of VGSC protein expression was investigated by immunocytochemistry, confocal microscopy and digital imaging (Fig. 5). This analysis showed that plasma membrane VGSC protein expression increased by 260 ± 3.2 % (p = 0.01; n = 18; Fig. 5C). In contrast, there was no significant change in the VGSC immunoreactivity of the intracellular compartment (Fig. 5C).

It was concluded that EGF increased Nav1.7 mRNA expression, leading to *de novo *VGSC protein synthesis, directed mainly to plasma membrane.

### Auto-regulation of VGSC protein expression

**Figure 6:**
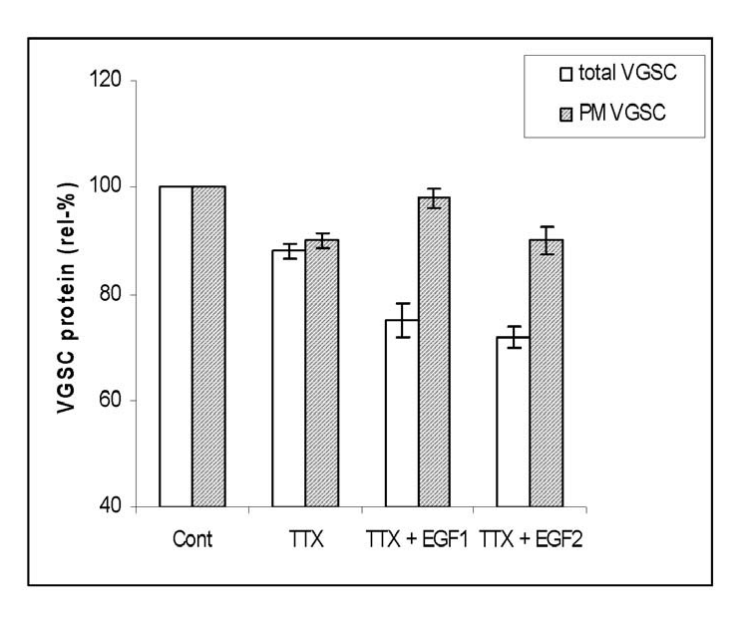
Effects of TTX (500 nM) and TTX+EGF on respective relative levels of total and plasma membrane (PM) VGSC protein expression, presented as a percentages of respective controls (Cont). Two concentrations of EGF were used: 50 ng/ml (EGF1) and 100 ng/ml (EGF2). Relative levels of total VGSC were deduced from Western blots (as in Fig. 2). Relative levels of PM expression were obtained from immunocytochemistry/digital analysis(as in Fig. 4). Each histobar denotes mean ± standard error (n = 3–6). Light bars, total VGSC protein. Shaded bars, VGSC protein expressed in plasma membrane (PM).

Taking together the data obtained here (using the strongly metastatic human PCa PC-3M cell line) and the data obtained previously from analogous rat PCa Mat-LyLu cells [10] (both with Nav1.7 dominant), VGSC expression would appear to be under auto-regulation. Accordingly, long-term (24+ h) treatment with TTX led to down-regulation of functional VGSC expression. Thus, application of TTX (500 nM) alone reduced total and plasma membrane VGSC protein expressions by 12 ± 1.5 % and 10 ± 1.4 %, respectively (p < 0.01 for both; n = 5–6; Fig. 6). Interestingly, even when EGF (50 and 100 ng/ml) was added in the presence of TTX, protein expressions remained below control levels. Thus, the auto-regulation by positive feed-back appeared to dominate the normally potentiating effect of EGF.

## Discussion

The main results of the present study are as follows: 1) EGF increased VGSC mRNA and protein expression; the latter was largely targeted to the plasma membrane. (2) Exogenous EGF enhanced PC-3M cells' *in vitro *metastatic behaviours (migration, endocytosis and invasion). Endogenous EGF appeared to have a similar involvement. (3) The potentiating effects of EGF on MCBs were mediated substantially by VGSC activity. (4) VGSC expression also was under auto-regulation by activity-dependent positive feed-back which dominated the effect of EGF.

### Role of EGF in PCa

Prostate cancer is extremely heterogeneous in stage and grade of tumours within individual glands and this adds to the complexity of its diagnosis [24]. As regards therapy, whilst androgen ablation works for a few years, hormone resistance ultimately sets in and this may coincide with PCa progression becoming dependent on growth factor signalling [25]. A key such growth factor may be EGF which has a well known role in embryogenesis, cellular differentiation, proliferation and angiogenesis. A high level of EGF immunoreactivity has been shown in normal rat prostate [26] and this may be controlled by androgen [27]. Androgen receptor can interact with EGFR signalling and this can regulate invasiveness [28]. In fact, the highest concentration of EGF in body (~175 ng/ml) occurs in prostate [29], comparable to the concentrations used in the present study. Upregulation of EGF/EGFR expression has been reported in metastatic PCa [30]. Exogenous EGF has been shown to enhance migration and invasion of various cell lines, including rat PCa Mat-LyLu cells [19]. The present study showed that EGF (exogenous and endogenous) enhanced the *in vitro *MCBs of the strongly metastatic PC-3M cells. Taken together, this evidence supports the notion that EGF signalling is closely associated with progression of PCa to the metastatic mode. Accordingly, EGF receptor antagonists or tyrosine kinase blockers are potential anti-PCa drugs [31].

### Regulation of functional VGSC expression by EGF

Our essential result and hypothesis to follow are that EGF induced upregulation of Nav1.7 mRNA and VGSC protein synthesis, most of which was inserted in plasma membrane and led to the observed enhancement of MCBs. We have shown previously for (rat and human) PCa cells that VGSC activity potentiates a range of cellular behaviours integral to the metastatic cascade, including morphological development [6], adhesion [9], transverse migration, endocytic membrane activity and invasion (present study; also, [10]). EGF-induced upregulation of VGSC activity has been shown previously in PC12 cells [17], and, more recently, in Mat-Lylu strongly metastatic rat PCa cells [19]. At present, the signalling cascades responsible for the observed transcriptional upregulation of VGSC in PC-3M cells is not known. There are 4 main transduction mechanism(s) associated with EGFR signalling: Stat, PLCγ, PI3K and MAPK [32]. It is possible that VGSC expression/activity was controlled at a variety of levels from transcription to post-translation, including mRNA stability and phosphorylation by PKC or PKA [e.g. [33]]. Further work is required to elucidate these aspects.

Although blocking VGSC activity with TTX suppressed EGF-induced enhancement of endocytosis and migration, the statistical analysis suggested that the involvement of VGSC in invasion was somewhat less. It is highly likely, in fact, that EGF would have parallel affects upon other functional cellular components involved in metastatic behaviour, including actin cytoskeleton [34], focal adhesion kinase [35] and Ca^2+ ^signalling [36]. A similar conclusion was reached from a similar study on rat PCa cells [19].

VGSC expression in PC-3M cells was also under auto-regulation by activity-dependent positive feed-back, such that blocking channel activity with TTX for 24 h would down-regulate its expression in plasma membrane. Such a mechanism was shown to occur in analogous strong metastatic rat PCa Mat-LyLu cells and involved PKA activated by the Na^+ ^influx occurring via active VGSCs [10]. The present study showed that a comparable feed-back mechanism also operates in human PCa cells, although it is not known if this also involves PKA as an intermediary. This auto-regulatory mechanism is robust and, if blocked, the up-regulatory effect of EGF was lost. It would follow, therefore, that EGF and VGSC are inter-connected in the regulation of functional VGSC expression and control of MCBs.

### Possible role of other growth factors and VGSC association in PCa

A number of other growth factors have also been associated with metastatic PCa. These include NGF [37], FGF [38], transforming growth factor-beta [39], vascular endothelial growth factor [40] and hepatocyte growth factor [41]. Some of these growth factors have also been shown to regulate VGSC activity e.g. [42]. The case of NGF is interesting since its concentration outside the nervous system is highest in the prostate. NGF was shown previously also to induce upregulation of VGSC expression/activity in PC12 cells [43], frog sympathetic B neurons [44], human astrocytoma cell lines (1321N1 and A172) [45] and rat PCa Mat-LyLu cells [10]. Importantly, however, although NGF also potentiated MCBs in Mat-LyLu cells, this did not occur via VGSC activity. It would seem, therefore, that the multitude of growth factors that occur in prostate may contribute to metastatic disease in different ways; this is likely to be a dynamic process and may be compartmentalised.

### EGF – VGSC interactions in PCa

Taking the available evidence, including the results of the present study together, a scheme of dual regulation VGSC expression/activity by EGF and feed-back can be considered for metastatic PCa, modelled here by PC-3M cells. In the main part, EGF is tonically released from PCa cells, consistent with the effect of AG1478 alone, also, [46,47]. This would upregulate VGSC expression/activity, thus potentiating MCBs. VGSC activity enhances secretory membrane activity in PCa cells, as indicated by the results of the endocytosis assays (also [8]). If VGSC activity controlled the release of EGF (eg via vesicular trafficking), it could lead to the following positive feed-back: VGSC activity → EGF release → VGSC upregulation etc. [48]. In a further loop, VGSC activity auto-regulates itself also by positive feed-back, as detailed previously for analogous rat PCa cells [10]. It is possible, in fact, that the two loops 'overlap' to some extent, and PKA may be a common factor [49]. Importantly, under conditions when VGSC activity was blocked, upregulation of VGSC protein expression (total or in plasma membrane) by EGF was not seen. It would follow, therefore that VGSC expression/activity is a key gate in progression of EGF-dependent (androgen-independent?) PCa. Accordingly, the metastatic process in PCa would be accelerated significantly by the two positive feed-back mechanisms (up)regulating VGSC expression. Further fine-tuning of this control mechanism may occur via interactions within the EGF system itself whereby, for example, EGF can control EGFR expression and vice versa, in autocrine fashion [50].

The present study highlighted further the potential of VGSC (Nav1.7) in clinical management of PCa [51]. We have shown previously that Nav1.7 expression has sufficient sensitivity and selectivity to be an effective diagnostic marker for metastatic PCa [14]. In fact, the biology of the VGSC (in being upregulated by growth factors, such as EGF, and enhancing various MCBs) is consistent with it being an early event in progression of PCa to metastasis. Suppression of VGSC activity directly using channel blockers, therefore, could have therapeutic potential. Indeed, VGSC-blocking anti-convulsant drugs have been shown to be cytostatic inhibitors of PCa [52]. The present work also raises the possibility of developing additionally effective combination therapy, aimed at concurrent blockage of VGSC activity and EGF signalling.

## Conclusion

The main conclusion is that EGF has a major involvement in the upregulation of functional VGSC expression in PC-3M human PCa cells. In turn, VGSC activity enhances the cells' *in vitro *migration, endocytosis and invasion. The effect of EGF is transcriptional, at least in part, and the *de novo *protein produced is largely targeted to the plasma membrane. VGSC expression is also under auto-regulation by activity-dependent positive feed-back. Thus, VGSC expression/activity could be a major intermediary of the potentiating effect of EGF in human PCa.

## Methods

### Cell culture and pharmacological treatments

PC-3M cells were maintained in RPMI medium (without phenol red) supplemented with 10 % foetal bovine serum (FBS) and 1 % L-glutamine, in a humidified 37°C incubator with 5 % CO_2 _. Prior to any pharmacological treatment, cells were first 'conditioned' in 0.5 % FBS for 24 h. 0.5 % FBS was determined as a optimum by testing the effect of a range of FBS concentrations on cell viability (data not shown). Cells were then incubated in one of the following: (1) EGF; (2) TTX; (3) EGF+TTX; (4) AG1478; an inhibitor of EGF receptor tyrosine kinase [53] or (5) EGF+AG1478. All pharmacological agents were applied in 0.5 % FBS for 24 h.

### *In vitro *metastatic cell behaviour assays

Initial 3-(4,5-Dimethylthiazol-2-yl)-2,5-diphenyltetrazolium bromide (MTT) assays showed that there was no proliferative effect of any of the pharmacological agents at their working concentrations. Lack of effect of EGF on proliferation of PCa cells has also been reported before [54-56]. Also, none of the treatments had any effect on cell viability, monitored using trypan blue staining. Three different assays of *in vitro *metastatic cell behaviour (MCB) were carried out, as follows:

1. *Transwell migration*. Details of this assay were described previously [57]. Essentially, conditioned cells seeded in multi-well dishes were pharmacologically treated and then re-suspended using trypsin-EDTA and plated at a density of 20 × 10^4 ^cells/well onto 12 μm pore Transwell filters with polycarbon membrane (Corning, MA, USA). Following 6 h incubation, MTT was used to determine the number of migrated cells. These measurements were plotted as the percentage (%) of the readings for migrated cells/original cell number, giving "Migration Index (MiI)".

2. *Endocytic membrane activity*. This followed the procedure originally described by Onganer and Djamgoz [58]. Briefly, cells were seeded in multi-well dishes at a density of 5 × 10^4 ^cells/well in normal medium. Endocytosis was measured by optimised (0.5 mg/ml for 40 min) uptake of horseradish peroxidase (HRP type IV; Sigma). Endogeneous peroxidase activity was measured in parallel in every experiment and subtracted from the uptake. The difference in the optical densities was assumed to represent true endocytosis and plotted as the percentage of the control level in untreated cells, giving "Endocytosis Index, EI (%)".

3. *Matrigel invasion*. Boyden invasion chambers with 8 μm pore size inserts pre-coated with Matrigel basement membrane matrix were used in accordance with the manufacturer's instructions (BD BioSciences, MA, USA) [4,57]. Cells were plated at a density 7.5 × 10^4 ^/well and each treatment was repeated three times. The optical density of the invaded cell population was measured after 24 h and plotted as a percentage of the original cell number, giving "Invasion Index, InvI (%)".

### Real-time PCR

Total RNA was isolated using a RNA miniprep kit according to the manufacturer's instructions (Stratagene, CA, USA). RNA quality was assessed by gel electrophoresis and its quantity was determined by spectrophotometric analysis. cDNA was generated by reverse transcriptase reaction and used for real time PCR (rt-PCR). The primer sequences for Nav1.7 were as follows:

5'-TATGACCATGAATAACCCGC-3'; and

5-'TCAGGTTTCCCATGAACAGC-3'

The annealing temperature is 60°C; [13]. Quantification of mRNA levels was performed by SYBR Green technology, using a DNA Engine Opticon 2 System (MJ Research). The mRNA levels were calculated by the 2^-ΔΔC^_T _method [59], with beta actin as the normalising gene [60]. Additional PCRs confirmed expression of EGF receptors in PC-3M cells (not shown) [23].

### Western blots

Proteins were extracted as described before [5] and prepared according to the instructions of the manufacturer (Upstate Biotechnology, Buckingham, UK). Protein yield was determined by spectrophotometry. The primary antibody was a pan-VGSC (Upstate Biotechnology, Buckingham, UK). The secondary antibody was a peroxidase-conjugated swine anti-rabbit immunoglobulin (DAKO, Glostrup, Denmark). The nitrocellulose membrane was stripped and treated with an anti-actinin antibody (Sigma, UK) as a loading control. For the latter, the secondary antibody was peroxidase-conjugated goat anti-mouse immunoglobulin (DAKO, Glostrup, Denmark). The amount of protein loaded has been specified in given figure legends. Optical density of the gels for all the treatments was calculated using by Image-Pro Plus software (Media Cybernetics).

### Cell surface biotinylation

Following pharmacological treatment, cells were washed three times with cold PBS. Live cells were incubated for 2 h with 1 mg/ml EZ-Link Sulfo-NHS-Biotin (Pierce, IL, USA) at 4°C. After washing with PBS containing 10 mM glycine, total cell protein was extracted, as before. One-half of the extracted protein was kept at -20°C for Western blots. The other half was labelled with streptavidin beads for 1 h. Beads were washed extensively and re-suspended in SDS sample buffer. Total and streptavin-labelled proteins were subject to Western blots, as described above.

### Immunocytochemistry and digital image analysis

Control or treated cells (2 × 10^4^) were seeded onto13 mm sterile coverslips (BHD, Poole, UK) pre-coated with poly-L-lysine in 24 well plates and fixed in 4 % PFA for 15 min. After washing (3 × 5 min with 0.1 % BSA in PBS; pH 7.4), plasma membranes were stained with concanavalin A for 45 min [10]. After further washing (×3), cells were permeabilised with 0.1 % saponin for 3 min, washed and blocked with 5 % BSA in PBS for 1 h at room temperature. The primary antibody, pan-VGSCα (Upstate Biotechnology, Buckingham, UK), diluted in 5 % BSA in PBS to the working concentration (1 μg/100 μl), was applied for 1 h at room temperature in a moist chamber and then washed off. The secondary antibody (a peroxidase-conjugated swine anti-rabbit immunoglobulin; DAKO, Glostrup, Denmark) and Alexaflour 568 were applied for 1 h at room temperature and then washed off. Finally, cells were washed in distilled H_2_O and mounted with anti-fading medium, Vectashield (Vector, Burlingame, USA). Cells were examined by confocal microscopy (Leica DM IRBE). Exposures and optical sectioning were identical for all the treatments.

VGSC protein expression in plasma membrane was quantified as the mean optical intensity of immunoreactivity of permeabilized cell outlines, calculated using the "freeform line profile" function (Lecia confocal software LCS Lite; version 2.00) drawn around the cell surface, determined by concanavalin A staining. Randomly chosen 50 cells (from 3 repeats in each experiment) were analysed. The subcellular distribution of VGSC protein was determined using the "straight line profile" function drawn across the cytoplasm avoiding the nucleus, as described previously [10]. Digitised optical density in plasma membrane region, set to cover 1.5 μm inward from the edge of concanavalin A staining, was compared with cytoplasmic signal density within the central 30 % of the line profile. Measurements were taken from randomly chosen 6 cells per condition for three repeat experiments.

### Data analysis

All data were analysed as means ± standard errors. Statistical significance was determined using Student's *t*-test or ANOVA with Newman-Keuls post-hoc analysis were used, as appropriate. Results were considered significant for p < 0.05.

## Abbreviations

VGSC: Voltage-gated sodium channel

EGF: Epidermal growth factor

TTX: Tetrodotoxin

PCa: Prostate cancer

MCB: Metastatic cell behaviour

## Competing interests

The author(s) declare that they have no competing interests.

## Authors' contributions

PUO drafted the manuscript, performed statistical studies, and carried out all experiments described in this paper, including Western blots, RNA extractions, PCRs, immunocytochemistry, functional assays (invasion, migration, endocytosis and proliferation). MBAD conceived the study, participated in its design and helped in data analyses and drafting and editing of the manuscript. Both authors read and approved the final manuscript.

## References

[B1] Nomura AM, Kolonel LN (1991). Prostate cancer: a current perspective. Epidemiol Rev.

[B2] Penson DF, Albertsen PC (2002). Lessons learnt about early prostate cancer from large scale databases: population-based pearls of wisdom. Surg Oncol.

[B3] Djamgoz MBA (1998). Voltage-gated sodium channel activity and metastasis: A novel approach to understanding the pathophysiology of prostate cancer. J Physiol.

[B4] Grimes JA, Fraser SP, Laniado ME, Foster CS, Abel PD, Djamgoz MBA (1995). Differential expression of voltage-activated Na^+ ^currents in two prostatic tumour cell lines: contribution to invasiveness in vitro. FEBS Lett.

[B5] Laniado ME, Lalani EN, Fraser SP, Grimes JA, Bhangal G, Djamgoz MB, Abel PD (1997). Expression and functional analysis of voltage-activated Na^+ ^channels in human prostate cancer cell lines and their contribution to invasion in vitro. Am J Pathol.

[B6] Fraser SP, Ding Y, Liu A, Foster CS, Djamgoz MBA (1999). Tetrodotoxin suppresses morphological enhancement of the metastatic MAT-LyLu rat prostate cancer cell line. Cell Tissue Res.

[B7] Djamgoz MBA, Mycielska ME, Madeja Z, Fraser SP, Korohoda W (2001). Directional movement of rat prostate cancer cells in direct-current electric field: Involvement of voltage-gated Na^+ ^channel activity. J Cell Sci.

[B8] Mycielska M, Fraser SP, Szatkowski M, Djamgoz MBA (2003). Contribution of functional voltage-gated Na^+ ^channel expression to cell behaviours involved in the metastatic cascade in rat prostate cancer. II Secretory membrane activity. J Cell Physiol.

[B9] Palmer CP, Mycielska ME, Burcu H, Osman K, Collins T, Beckerman R, Perrett R, Aydar E, Djamgoz MBA (2007). A micro-pressure system for measuring single cell adhesion: application to cancer cell lines of different metastatic potential and voltage-gated Na^+ ^channel expression. Eur Biophys J.

[B10] Brackenbury WJ, Djamgoz MB (2006). Activity-dependent regulation of voltage-gated Na^+ ^channel expression in Mat-LyLu rat prostate cancer cell line. J Physiol.

[B11] Smith P, Rhodes NP, Shortland AP, Fraser SP, Djamgoz MBA, Ke Y, Foster CS (1998). Sodium channel protein expression enhances the invasiveness of rat and human prostate cancer cells. FEBS Lett.

[B12] Bennett ES, Smith BA, Harper JM (2004). Voltage-gated Na^+ ^channels confer invasive properties on human prostate cancer cells. Pflugers Arch.

[B13] Diss JK, Archer SN, Hirano J, Fraser SP, Djamgoz MBA (2001). Expression profiles of voltage-gated Na^+ ^channel α-subunit genes in rat and human prostate cancer cell lines. Prostate.

[B14] Diss JK, Stewart D, Pani F, Foster CS, Walker MM, Patel A, Djamgoz MB (2005). A potential novel marker for human prostate cancer: voltage-gated sodium channel expression in vivo. Prostate Cancer Prostatic Dis.

[B15] Lou JY, Laezza F, Gerber BR, Xiao M, Yamada KA, Hartmann H, Craig AM, Nerbonne JM, Ornitz DM (2005). Fibroblast growth factor 14 is an intracellular modulator of voltage-gated sodium channels. J Physiol.

[B16] Lei S, Dryden WF, Smith PA (2001). Nerve growth factor regulates sodium but not potassium channel currents in sympathetic B neurons of adult bullfrogs. Neurophysiol.

[B17] Toledo-Aral JJ, Brehm P, Halegoua S, Mandel G (1995). A single pulse of nerve growth factor triggers long-term neuronal excitability through sodium channel gene induction. Neuron.

[B18] Brackenbury WJ, Djamgoz MB (2007). Nerve growth factor enhances voltage-gated Na channel activity and Transwell migration in Mat-LyLu rat prostate cancer cell line. J Cell Physiol.

[B19] Ding Y, Brackenbury WJ, Onganer PU, Montano X, Porter L, Bates LF, Djamgoz MBA (2007). Epidermal growth factor upregulates motility of Mat-LyLu rat prostate cancer cells partially via voltage-gated sodium channel activity. J Cell Physiol.

[B20] Culig Z, Hobisch A, Cronauer MV, Radmayr C, Trapman J, Hittmair A, Bartsch G, Klocker H (1996). Androgen receptor activation in prostatic tumor cell lines by insulin-like growth factor-I, keratinocyte growth factor, and epidermal growth factor. Cancer Res.

[B21] Delongchamps NB, Peyromaure M, Dinh-Xuan AT (2006). Role of vascular endothelial growth factor in prostate cancer. Urology.

[B22] Hakariya T, Shida Y, Sakai H, Kanetake H, Igawa T (2006). EGFR signaling pathway negatively regulates PSA expression and secretion via the PI3K-Akt pathway in LNCaP prostate cancer cells. Biochem Biophys Res Commun.

[B23] Greene GF, Kitadai Y, Pettaway CA, von Eschenbach AC, Bucana CD, Fidler IJ (1997). Correlation of metastasis-related gene expression with metastatic potential in human prostate carcinoma cells implanted in nude mice using an in situ messenger RNA hybridization technique. Am J Pathol.

[B24] Arora R, Koch MO, Eble JN, Ulbright TM, Li L, Cheng L (2004). Heterogeneity of Gleason grade in multifocal adenocarcinoma of the prostate. Cancer.

[B25] Roznovanu SL, Amalinci C, Radulescu D (2005). Molecular mechanisms in hormone-resistant prostate cancer. Rev Med Chir Soc Med Nat Iasi.

[B26] Torring N, Jorgensen PE, Poulsen SS, Nexo E (1998). Epidermal growth factor in the rat prostate: production, tissue content and molecular forms in the different prostatic lobes. Prostate.

[B27] Gregory J, Willshire IR, Kavanagh JP, Blacklock NJ, Chowdury S, Richards RC (1986). Urogastrone-epidermal growth factor concentrations on prostatic fluid of normal individuals and patients with benign prostatic hypertrophy. Clin Sci.

[B28] Bonaccorsi L, Muratori M, Carloni V, Marchiani S, Formigli L, Forti G, Baldi E (2004). The androgen receptor associates with the epidermal growth factor receptor in androgen-sensitive prostate cancer cells. Steroids.

[B29] Gann PH, Klein KG, Chatterton RT, Ellman AE, Grayhack JT, Nadler RB, Lee C (1999). Growth factors in expressed prostatic fluid from men with prostate cancer, BPH, and clinically normal prostates. Prostate.

[B30] De Miguel P, Royuela, Bethencourt R, Ruiz A, Fraile B, Paniagua R (1999). Immunohistochemical comparative analysis of transforming growth factor alpha, epidermal growth factor, and epidermal growth factor receptor in normal, hyperplastic and neoplastic human prostates. Cytokine.

[B31] Hegeman RB, Liu G, Wilding G, McNeel DG (2004). Newer therapies in advanced prostate cancer. Clin Prostate Cancer.

[B32] Oda K, Matsuoka Y, Funahashi A, Kitano H (2005). A comprehensive pathway map of epidermal growth factor receptor signaling. Mol Syst Biol.

[B33] Hilborn MD, Rane SG, Pollock JD (1997). EGF in combination with depolarization or cAMP produces morphological but not physiological differentiation in PC12 cells. J Neurosci Res.

[B34] Toral C, Solano-Agama C, Reyes-Marquez B, Sabanero M, Talamas P, Gonzalez Del Pliego M, Mendoza-Garrido ME (2007). Role of extracellular matrix-cell interaction and epidermal growth factor (EGF) on EGF-receptors and actin cytoskeleton arrangement in infantile pituitary cells. Cell Tissue Res.

[B35] Tapia JA, Camello C, Jensen RT, Garcia LJ (1999). EGF stimulates tyrosine phosphorylation of focal adhesion kinase (p125FAK) and paxillin in rat pancreatic acini by a phospholipase C-independent process that depends on phosphatidylinositol 3-kinase, the small GTP-binding protein, p21rho, and the integrity of the actin cytoskeleton. Biochim Biophys Acta.

[B36] Chan AS, Wong YH (2004). Epidermal growth factor differentially augments G(i)-mediated stimulation of c-Jun N-terminal kinase activity. Br J Pharmacol.

[B37] Djakiew D, Delsite R, Pflug B, Wrathall J, Lynch JH, Onoda M (1991). Regulation of growth by a nerve growth factor-like protein which modulates paracrine interactions between a neoplastic epithelial cell line and stromal cells of the human prostate. Cancer Res.

[B38] Pienta KJ, Isaacs WB, Vindivich D, Coffey DS (1991). The effects of basic fibroblast growth factor and suramin on cell motility and growth of rat prostate cancer cells. J Urol.

[B39] Barrack ER (1997). TGF beta in prostate cancer: a growth inhibitor that can enhance tumorigenicity. Prostate.

[B40] Balbay MD, Pettaway CA, Kuniyasu H, Inoue K, Ramirez E, Li E, Fidler IJ, Dinney CP (1999). Highly metastatic human prostate cancer growing within the prostate of athymic mice overexpresses vascular endothelial growth factor. Clin Cancer Res.

[B41] Nishimura K, Kitamura M, Takada S, Nonomura N, Tsujimura A, Matsumiya K, Miki T, Matsumoto K, Okuyama A (1998). Regulation of invasive potential of human prostate cancer cell lines by hepatocyte growth factor. Int J Urol.

[B42] Rush AM, Wittmack EK, Tyrrell L, Black JA, Dib-Hajj SD, Waxman SG (2006). Differential modulation of sodium channel Na(v)1.6 by two members of the fibroblast growth factor homologous factor 2 subfamily. Eur J Neurosci.

[B43] Bouron A, Becker C, Porzig H (1999). Functional expression of voltage-gated Na^+ ^and Ca^2+ ^channels during neuronal differentiation of PC12 cells with nerve growth factor or forskolin. Naunyn Schmiedebergs Arch Pharmacol.

[B44] Lei S, Dryden WF, Smith PA (2001). Nerve growth factor regulates sodium but not potassium channel currents in sympathetic B neurons of adult bullfrogs. J Neurophysiol.

[B45] Kraft R, Basrai D, Benndorf K, Patt S (2001). Serum deprivation and NGF induce and modulate voltage-gated Na currents in human astrocytoma cell lines. Glia.

[B46] Connolly JM, Rose DP (1990). Production of epidermal growth factor and transforming growth factor-alpha by the androgen-responsive LNCaP human prostate cancer cell line. Prostate.

[B47] Connolly JM, Rose DP (1991). Autocrine regulation of DU145 human prostate cancer cell growth by epidermal growth factor-related polypeptides. Prostate.

[B48] Montano X, Djamgoz MB (2004). Epidermal growth factor, neurotrophins and the metastatic cascade in prostate cancer. FEBS Lett.

[B49] Fassett J, Tobolt D, Hansen LK (2006). Type I collagen structure regulates cell morphology and EGF signaling in primary rat hepatocytes through cAMP-dependent protein kinase A. Mol Biol Cell.

[B50] Normanno N, De Luca A, Bianco C, Strizzi L, Mancino M, Maiello MR, Carotenuto A, De Feo G, Caponigro F, Salomon DS (2006). Epidermal growth factor receptor (EGFR) signaling in cancer. Gene.

[B51] Roger S, Potier M, Vandier C, Besson P, Le Guennec JY (2006). Voltage-gated sodium channels: new targets in cancer therapy?. Curr Pharm Des.

[B52] Anderson JD, Hansen TP, Lenkowski PW, Walls AM, Choudhury IM, Schenck HA, Friehling M, Holl GM, Patel MK, Sikes RA, Brown ML (2003). Voltage-gated sodium channel blockers as cytostatic inhibitors of the androgen-independent prostate cancer cell line PC-3. Mol Cancer Ther.

[B53] Zhu XF, Liu ZC, Xie BF, Li ZM, Feng GK, Yang D, Zeng YX (2001). EGFR tyrosine kinase inhibitor AG1478 inhibits cell proliferation and arrests cell cycle in nasopharyngeal carcinoma cells. Cancer Lett.

[B54] Chopra DP, Grignon DJ, Joiakim A, Mathieu PA, Mohamed A, Sakr WA, Powell IJ, Sarkar FH (1996). Differential growth factor responses of epithelial cell cultures derived from normal human prostate, benign prostatic hyperplasia, and primary prostate carcinoma. J Cell Physiol.

[B55] Jones HE, Eaton CL, Barrow D, Dutkowski CM, Gee JM, Griffiths K (1997). Comparative studies of the mitogenic effects of epidermal growth factor and transforming growth factor-alpha and the expression of various growth factors in neoplastic and non-neoplastic prostatic cell lines. Prostate.

[B56] Guo C, Luttrell LM, Price DT (2000). Mitogenic signaling in androgen sensitive and insensitive prostate cancer cell lines. J Urol.

[B57] Fraser SP, Diss JK, Chioni AM, Mycielska ME, Pan H, Yamaci RF, Pani F, Siwy Z, Krasowska M, Grzywna Z, Brackenbury WJ, Theodorou D, Koyuturk M, Kaya H, Battaloglu E, De Bella MT, Slade MJ, Tolhurst R, Palmieri C, Jiang J, Latchman DS, Coombes RC, Djamgoz MB (2005). Voltage-gated sodium channel expression and potentiation of human breast cancer metastasis. Clin Cancer Res.

[B58] Onganer PU, Djamgoz MBA (2005). Small-cell lung cancer (human): potentiation of endocytic membrane activity by voltage-gated Na channel expression in vitro. J Membr Biol.

[B59] Livak KJ, Schmittgen TD (2001). Analysis of relative gene expression data using real-time quantitative PCR and the 2(-Delta Delta C(T)) Method Methods.

[B60] Aydar E, Onganer P, Perrett R, Djamgoz MB, Palmer CP (2006). The expression and functional characterization of sigma (sigma) 1 receptors in breast cancer cell lines. Cancer Lett.

